# Expression of chicken interleukin-2 by a highly virulent strain of Newcastle disease virus leads to decreased systemic viral load but does not significantly affect mortality in chickens

**DOI:** 10.1186/s12985-015-0353-x

**Published:** 2015-08-08

**Authors:** Leonardo Susta, Diego G. Diel, Sean Courtney, Stivalis Cardenas-Garcia, Roy S. Sundick, Patti J. Miller, Corrie C. Brown, Claudio L. Afonso

**Affiliations:** USDA ARS, Southeast Poultry Research Laboratory, 934 College Station Rd, Athens, GA 30605 USA; Department of Veterinary Pathology, College of Veterinary Medicine, University of Georgia, Athens, GA 30605 USA; Department of Immunology and Microbiology, Wayne State University, Detroit, MI 48201 USA; Present address: Department of Pathobiology, Ontario Veterinary College, University of Guelph, Guelph, ON N1G 2W1 Canada; Present address: Department of Veterinary and Biomedical Sciences, College of Agriculture and Biological Sciences, South Dakota State University, Brookings, SD 57007 USA

## Abstract

**Background:**

In mammals, interleukin 2 (IL-2) has been shown to decrease replication or attenuate pathogenicity of numerous viral pathogens (herpes simplex virus, vaccinia virus, human respiratory syncytial virus, human immunodeficiency virus) by activating natural killer cells (NK), cytotoxic T lymphocytes and expanding subsets of memory cells. In chickens, IL-2 has been shown to activate T cells, and as such it might have the potential to affect replication and pathogenesis of Newcastle disease virus (NDV).

**Methods:**

To assess the effect of IL-2 during NDV infection in chickens, we produced a recombinant virulent NDV strain expressing chicken IL-2 (rZJ1-IL2). The effects of IL-2 expression were investigated *in vivo* using the intracerebral pathogenicity index (ICPI) in day-old chicks and pathogenesis experiments in 4-week-old chickens. In these studies, rZJ1-IL2 was compared to a control virus expressing the green fluorescent protein (rZJ1-GFP). Assessed parameters included survival curves, detailed histological and immunohistochemical grading of lesions in multiple organs, and virus isolation in blood, spleen and mucosal secretions of infected birds.

**Results:**

At the site of infection (eyelid), expression of IL-2 was demonstrated in areas of rZJ-IL2 replication, confirming IL-2 production *in vivo*. Compared to rZJ1-GFP strain, rZJ1-IL2 caused milder lesions and displayed decreased viral load in blood, spleen and mucosal secretions of infected birds. In the rZJ1-IL2-infected group, virus level in the blood peaked at day 4 post-infection (pi) (10^3.46^ EID_50_ /0.1 ml) and drastically decreased at day 5 pi (10^0.9^ EID_50_/0.1 ml), while in the rZJ1-GFP-infected group virus levels in the blood reached 10^5.35^ EID_50_/0.1 ml at day 5. However, rZJ1-IL2-infected groups presented survival curves similar to control birds infected with rZJ1-GFP, with comparable clinical signs and 100 % mortality. Further, expression of IL-2 did not significantly affect the ICPI scores, compared to rZJ1-GFP strain.

**Conclusions:**

Increased expression of chicken IL-2 during virulent NDV replication in naïve chickens decreased viral titers in blood, spleens, oral and cloacal secretions on day 4–5 post infection. This is consistent with the previously described role of IL-2 in enhancing the clearance of viruses in mammals, such as human respiratory syncytial virus.

**Electronic supplementary material:**

The online version of this article (doi:10.1186/s12985-015-0353-x) contains supplementary material, which is available to authorized users.

## Background

Newcastle disease (ND) severely affects poultry worldwide and causes severe economic losses both in developing and developed countries [1]. The disease is caused by virulent strains of Newcastle disease virus (NDV), an enveloped virus classified in the *Mononegavirales* order, *Paramyxoviridae* family, *Avulavirus* genus [2]. NDV strains have a non-segmented, negative sense RNA genome of approximately 15.2 kb, which encodes for at least seven proteins, namely nucleoprotein (NP), phosphoprotein (P), V protein (transcribed by post-transcriptional modification of the P mRNA), matrix (M), hemagglutinin neuraminidase (HN), fusion (F), and the large polymerase (L) [3]. According to international standards, NDV strains can be classified as virulent or non-virulent, based on the intracerebral pathogenicity index (ICPI), and the amino acid sequence of the F protein [4]. A NDV strain is considered virulent if it has an ICPI greater than or equal to 0.7, or if the F protein has at least three basic amino acids from residues 113 to 116 and a phenylalanine at position 117 [3–5].

The ability to cause clinical signs and lesions varies greatly among NDV strains [6]. Clinically, NDV strains have been classified as highly virulent (velogenic), moderately virulent (mesogenic), and non-virulent (lentogenic and asymptomatic enteric strains). Velogenic strains are divided into viscerotropic, if they cause severe necro-hemorrhagic lesions in visceral organs, or neurotropic if they cause mainly neurological disease [3, 6].

Based on transcriptional analysis studies, infection with virulent strains of NDV induces transcriptional upregulation of numerous cytokines, such as interferon alpha (IFN-α), interferon gamma (IFN-γ), interleukin 8 (IL-8), and interleukin 2 (IL-2) [7–10]. It is speculated that these cytokines might modulate the immune response during infection. It remains unclear, however, if their expression contributes to NDV-induced tissue damage and mortality (similar to the “cytokine storm” seen in mammals [11–14]), or represents an inadequate response to control virus replication and limit tissue damage.

In mammals, IL-2 facilitates clearance and decreases pathogenicity of numerous viral pathogens. In mice, a recombinant human herpes simplex-1 virus (HSV-1) expressing IL-2 showed decreased mortality compared to the wild-type parental strain, and this protection was linked to the activity of effector CD4+ and CD8+ lymphocytes [15]. Similarly, a IL-2 double knock-out mice inoculated with HSV-1 in the conjunctiva showed higher mortality, increased magnitude of virus replication in the eye, and more pronounced blepharitis compared to IL-2-competent mice [16]. Treatment of BALB/c mice with recombinant IL-2 decreased replication of murine cytomegalovirus (CMV) *in vivo* [17]. When cloned into the genome of the murine poxvirus (Ectromelia virus), expressed IL-2 was able to decrease the pathogenicity of this virus in athymic nude mice, most likely by inducing production of IFN-γ by natural killer (NK) cells [18, 19]. Most significantly, expression of IL-2 by the respiratory paramyxovirus human respiratory syncytial virus (hRSV) increased virus clearance in mice [20]. In humans, IL-2 was associated with non-cytolytic clearance of human immunodeficiency virus (HIV) in CD4+ cells [21].

Chicken IL-2 was first cloned in 1997 [22, 23]. In avian species, IL-2 has effects comparable to mammals, including lymphocyte proliferation, activation of NK cells, and clearance of intracellular pathogens [24, 25]. Moderate upregulation of IL-2 has been observed in tissues of chickens infected with virulent NDV strains [8–10], however little is known about the effect of IL-2 on the virulence of NDV, and it remains unclear if IL-2 might have an antiviral effect against this virus. Given the demonstrated antiviral effects of IL-2 against several mammalian viruses, and the pleiotropic effects that IL-2 has in the immune response (some of which have antiviral activity, such as promoting a Th1-biased immune response and cell-mediated immunity), it is possible that IL-2 might contribute to a faster clearance of NDV during infection. To investigate the hypothesis that IL-2 has an antiviral effect against NDV *in vivo*, we constructed a velogenic NDV that expresses chicken IL-2 during viral replication, and we evaluated its effect in 4-week-old chickens.

## Results

### Production and rescue of recombinant viruses

The full-length clone (FLC) plasmid of the virulent wild-type ZJ1 NDV strain was used to introduce the open-reading frame (ORF) of chicken IL-2, between the P and M genes, as previously described [26]. As a control for the increased genome size, the ORF of the enzymatically inactive green fluorescent protein (GFP) was inserted in the FLC plasmid at the same genomic location. In order to rescue the recombinant viruses, FLC plasmids containing the ORF of IL-2, GFP, or no insert were transfected in Hep-2 cells together with helper plasmids (NP, P and L genes from the NDV LaSota strain) in order to rescue rZJ1-IL2, rZJ1-GFP, and rZJ1, respectively. Recombinant viruses were successfully rescued in Hep-2 cells and propagated with two to three passages in 10-day old specific pathogen free (SPF) embryonated chicken eggs. RNA was extracted from the allantoic fluid of infected eggs, and to confirm the identity of the virus, the region corresponding to the insertion site (nucleotides 2857–3676 of the rZJ1 genome) was amplified by reverse transcriptase PCR (RT-PCR) and sequenced to assess the presence of the inserted sequences (see Material and Methods).

### High levels of IL2 are produced by rZJ1-IL2 in both *in vitro* and *in vivo*

**Fig. 1:**
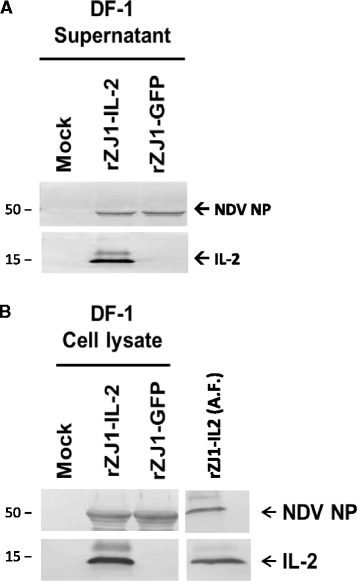
Production of chicken IL-2 was assessed by western blot *in vitro* (DF-1 cell line) and *in vivo* (infected eggs) at 48 hpi. A strong band of approximately 15 kDa is present in blots from the supernatant (panel **a**), cell lysate (panel **b**), and allantoic fluid (panel **b**) of rZJ1-IL2-infected DF-1 cells and eggs. No band of the same molecular weight was present in blots from the rZJ1-GFP-infected groups. Blots from the supernatant (panel **a**), cell lysates (panel **b**), and allantoic (panel **b**) fluid of both rZJ1-IL2 and rZJ1-GFP groups showed bands for NDV NP at approximately 50 kDa. No bands were observed in mock-inoculated controls, either at 15 or 50 kDa

Concurrent expression of IL-2 and NDV NP was assessed both *in vitro* (DF-1 cells) and *in vivo* (embryonated eggs) by western blot (WB) (see Material and Methods). Immortalized chicken embryo fibroblasts (DF-1 cells) and 9 to 10-day embryonated SPF chicken eggs were infected with rZJ1-IL2, rZJ1-GFP, or simple media (control). At 48 h post-infection (hpi), cell supernatant, cells lysate, and allantoic fluid were harvested and assayed using WB. A band of approximately 15 KDa, corresponding to the molecular weight of chicken IL-2 [24] and possibly the glycosylated form of IL2, were detected from the supernatant and cell extracts of DF-1 cells infected with rZJ1-IL2, but not rZJ1-GFP, or in the control group (Fig. 1a and b). A band of approximately 53 KDa, corresponding to the predicted molecular weight of NDV NP [27], was detected from the supernatant and cell extracts of DF-1 cells infected with rZJ1-IL2 and rZJ1-GFP, but not in the control group (Fig. 1a and b). Similarly, strong bands of 15 KDa and 53 KDa were observed from the allantoic fluid of rZJ1-IL2-infected eggs (Fig. 1b). Blots from allantoic fluid of rZJ1-GFP-infected eggs showed the 53 KDa but no the 15 KDa bands, whereas control eggs (non-infected) were negative for both bands (data not shown). Taken together these data show that, while both rZJ1-IL2 and rZJ1-GFP replicate in cell cultures and eggs, only rZJ1-IL2 expresses chicken IL-2 at high quantities both *in vitro* and *in vivo*.

### Expression of IL-2 does not affect NDV replication in DF-1 or HD-11 cells

**Fig. 2:**
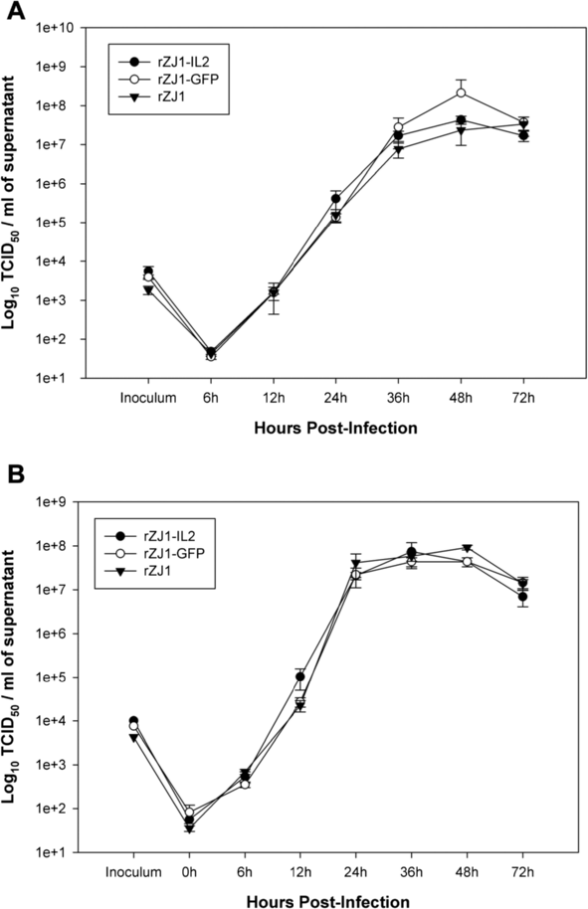
Multi-step growth kinetics of rZJ1-IL2, rZJ1-GFP, and rZJ1 in immortalized chicken fibroblast cell line (DF-1, panel **a**), and immortalized chicken macrophage cell line (HD-11, panel **b**). DF-1 and HD-11 cells were infected with each virus at a MOI = 0.01. At each time point, samples of supernatant were collected and titered by limiting dilutions in DF-1 cells (expressed as TCID_50_ / ml). Results show that there are not significant differences (*Two-way ANOVA*) in the growth of the three viruses in either cell line. Bars represent standard error (SE), *n* = 3

To assess the effect of chicken IL-2 production on the growth characteristics of rZJ1-IL2 and to verify that insertion of a foreign gene had not caused delay in rZJ1-IL2 growth, multi-step growth curves were carried out with rZJ1-IL2, rZJ1-GFP and rZJ1 in immortalized chicken embryo fibroblast (DF-1) and immortalized chicken macrophage cell lines (HD-11) (Fig. 2a and b). HD-11 cells were chosen because they may be biologically more relevant than DF-1 cells, due to the preferential tropism of virulent NDV strains for mononuclear phagocytic cells [6]. Briefly, cells were plated at 1×10^6^ cells/well (DF-1) or 7×10^5^ cells/well (HD-11) into 6-well plates, and were infected the next day with each NDV strain at a MOI = 0.01. Supernatant was collected at 0, 6, 12, 24, 36, 48, and 72 hpi. Virus titers were assessed by limiting dilutions in 96-well plates in DF-1 cells and expressed as tissue culture infectious dose 50 % (TCID_50_), according to the Spearmann-Karber method [28]. Statistical analysis (*Two-way ANOVA*, and *Tukey test for multiple comparisons*) demonstrated that virus titers at each time point did not differ between viruses (*p* = 0.268 and *p* = 0.1426 for the effect of virus strain in DF-1 and HD-11 cells, respectively), indicating that rZJ1-IL2, rZJ1-GFP, and rZJ1 had comparable replication kinetics and that IL-2 expression did not affect replication of rZJ1 in DF-1 or HD-11 cells (Fig. 2a and b). In DF-1 cells, highest virus yields (mean ± SD, expressed as log_10_ TCID_50_ units / ml) were 7.63 (+0.9, −0.11), 8.36 (+0.4, −0.4), and 7.71 (+0.18, −0.31) for rZJ1-IL2, rZJ1-GFP, and rZJ1 respectively (Fig. 2a). In HD-11 cells, highest yields were 7.87 (+0.2, −0.4), 7.63 (+0.09, −0.11), and 7.96 (+0.05, −0.05) (Fig. 2b).

### ICPI

The effect of IL2 expression on rZJ1 virulence was assessed *in vivo* using the intracerebral pathogenicity index (ICPI), which is the international standard to assess the virulence of NDV strains [4, 5]. The ICPI score for rZJ1-IL2 was 1.70, and the scores for rZJ1-GFP and rZJ1 have been previously published by our laboratory (using the same SPF White Leghorn flock) and are 1.64 and 1.85 [26]. These results indicate that IL-2 expression did not significantly change NDV virulence in day-old chickens, compared to the same virus expressing a gene with not known biological activity (GFP). Both rZJ1-IL2 and rZJ1-GFP have a lower ICPI score compared to the parental strain rZJ1 due to insertion of an additional transcriptional cassette [29, 30].

### Survival curves and clinical assessment

**Fig. 3:**
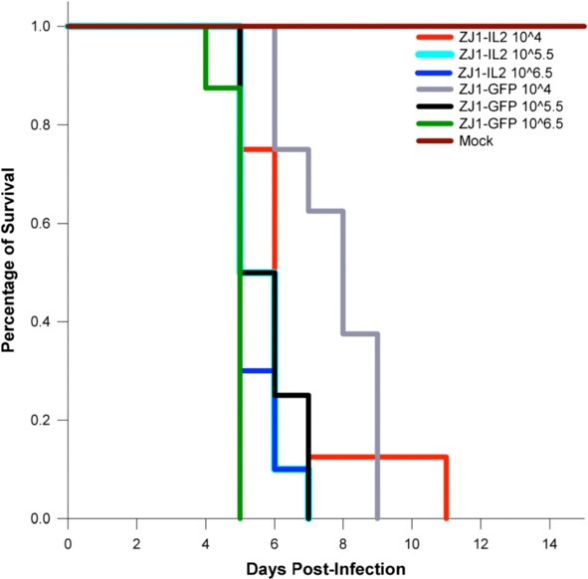
Survival curves in 4-week-old White Leghorn chickens illustrating mortality rates between rZJ1-IL2 and rZJ1-GFP-infected groups at three different doses of inoculum (low, 10^4^ TCID_50_ / bird; medium, 10^5.5^ TCID_50_ / bird; high, 10^6.5^ TCID_50_ / bird). Each color represents a different group at a different inoculum amount. No statistical differences were observed between rZJ1-IL2 or rZJ1-GFP groups within the same inoculum dose (*log-rank test*)

Although the ICPI test is broadly used to assess the pathogenicity of NDV strains, it is an artificial system in one day old chicks that does not always accurately predict the lesion-inducing ability of strains inoculated through a natural route of infection, nor it is comprehensive enough to estimate minor differences in virus replication or clinical signs in adult birds [6]. For this purpose, groups of 4-week-old White Leghorn chickens were inoculated (eyelid instillation) with rZJ1-IL2 or rZJ1-GFP at three different target doses: 10^4^ (low), 10^5.5^ (medium), 10^6.5^ (high) TCID_50_ / bird. Birds were monitored for clinical signs and deaths were recorded to produce a survival curve during 15 days (based on the expected time for all of the animals to die). Survival curves are presented in Fig. 3 and a detailed summary of the observed clinical signs is shown in Additional file 1: Table S1. Back-titers of virus inoculum were: 10^4.38^, 10^5.88^, and 10^6.25^ TCID_50_ / bird for rZJ1-IL2, and 10^3.75^, 10^5.5^, and 10^6.38^ TCID_50_ / bird for rZJ1-GFP.

**Fig. 4:**
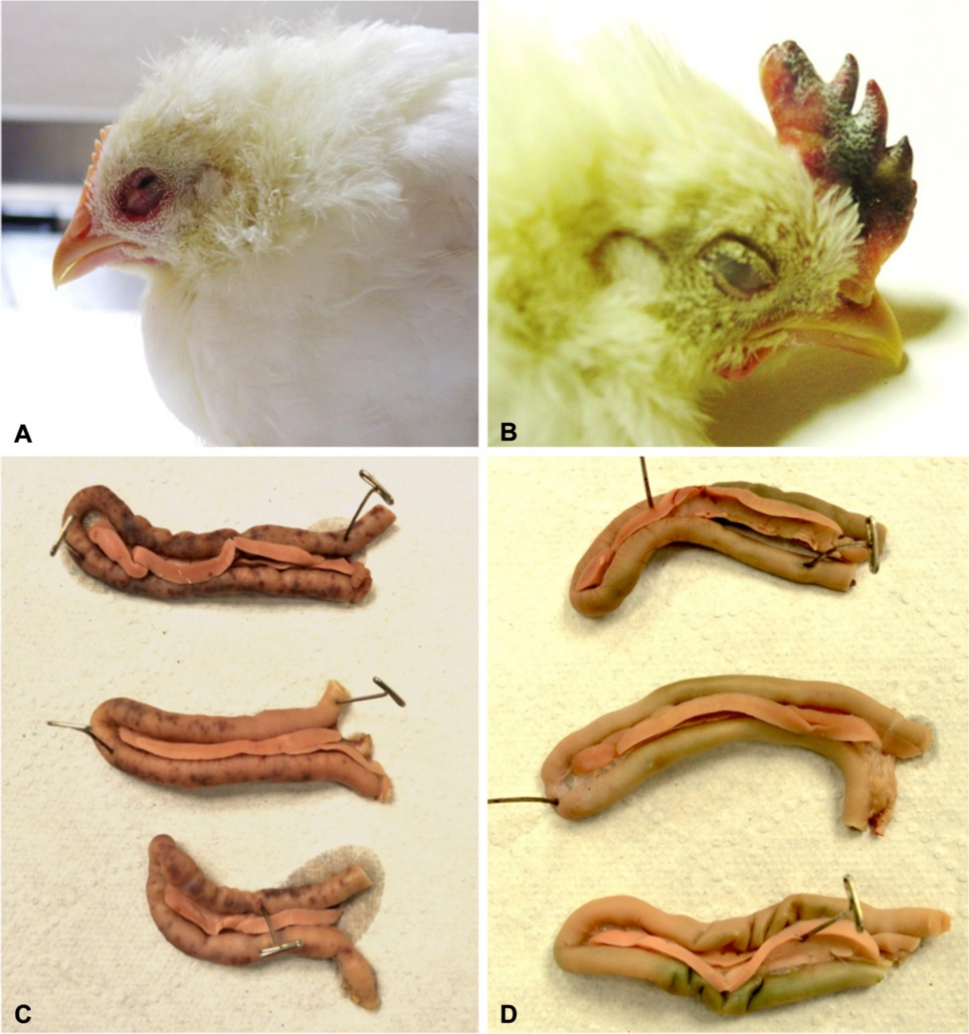
Macroscopic pictures illustrating lesions in rZJ1-IL2 and rZJ1-GFP-infected chickens. Panel **a**: eyelid; 4-week-old White Leghorn chicken infected with rZJ1-IL2, day 4 pi. Severe conjunctivitis characterized by hemorrhages, edema and congestion of the upper and lower eyelid. Panel **b**: comb; 4-week-old White Leghorn chickens infected with rZJ1-IL2, day 6 pi. Severe comb hemorrhages, edema, and cyanosis. Panel **c** and **d**: duodenal loop; 4-week-old White Leghorn chicken infected with rZJ1-GFP (**c**) and rZJ1-IL2 (**d**), day 4 pi. Note absence of intestinal hemorrhages in ZJ1-IL2-infected birds, compared to ZJ1-GFP-infected birds

Conjunctivitis was first observed at day 2 post-infection (pi) in birds infected with the high dose of rZJ1-IL2. Overall, conjunctivitis was more pronounced and severe in the rZJ1-IL2-infected birds than in the rZJ1-GFP-infected groups (Fig. 4, panel a). At day 4 and 5 pi, up to 13 % of the rZJ1-IL2-infected birds developed remarkably severe comb hemorrhages and edema (low and medium dose) (Fig. 4, panel b). Both in rZJ1-IL2 and rZJ1-GFP groups, birds developed depression and neurological signs by day 4 pi. Neurological signs consisted of head twitch, inability to stand, or hypermetric gait. Death patterns were as follows: for the low dose inoculation group, all rZJ1-IL2 and rZJ1-GFP-infected birds died at day 11 and 9 pi, respectively; for the medium dose inoculation group, all rZJ1-IL2 and rZJ1-GFP-infected birds died at day 7 pi; and for the high dose inoculation group, all rZJ1-IL2 and rZJ1-GFP-infected birds died at day 7 and 5 pi, respectively. Birds infected with rZJ1-IL2 took a longer time (48 h) overall to cause death of all the infected birds compared to rZJ1-GFP in the low and high dose infection groups, suggesting a protective effect of IL2, however no statistical differences in mortality (*log-rank test*, multiple comparisons) were observed between groups inoculated with the same virus concentrations (Fig. 3). Mock-infected birds showed no mortality or clinical signs.

### Pathogenesis experiment

A second animal experiment was conducted in order to assess in more details the ability of rZJ1-IL2 to cause lesions, and to evaluate its tissue distribution in key organs. To evaluate early development of gross and microscopic lesions, samples were taken during the first 5 days of infection. Groups of 20, 4-week-old chickens were inoculated (eyelid instillation) with either rZJ1-IL2 or rZJ1-GFP at a dose of 10^5.6^ and 10^6.2^ TCID_50_ / bird, respectively, as assessed by backtitration (target dose 10^5.5^). A BHI-inoculated group served as mock control. Birds were examined daily and each day 5 animals were used to collect swabs (oral and cloacal), blood, and spleens for virus isolation (VI) and titration. Three animals were necropsied at each time point for assessment of gross lesions and sampling of tissues for histopathology. Detailed summary of clinical signs and gross lesions is reported in Table 1.

**Table 1:** Summary of clinical signs and lesions in 4-week-old chickens infected with rZJ1-GFP, rZJ1-IL2, and BHI

Recombinant virus	Clinical signs	Lesions
rZJ1-GFP	Day 1 pi: no signs	Day 2 pi: splenomegaly (3/3); petechial hemorrhages in thymus (2/3).
	Day 2 pi: no signs	Day 3 pi: conjunctivitis (2/3); mottled and enlarged spleen (3/3).
	Day 3: mild conjunctivitis	Day 4 pi: conjunctivitis (1/3); thymic atrophy (2/3); enlarged mottled spleen (2/3); petechial hemorrhages diffuse in the intestine (3/3); necrosis in cecal tonsils (2/3); hemorrhages in proventriculus (1/3); bursal atrophy (1/3); severely dehydrated (1/3).
	Day 4 pi: moderate conjunctivitis and depression (3/10 had severe depression)	Day 5 pi: enlarged mottled spleen (3/3); hemorrhages in: duodenal loop, cecal tonsils, and throughout intestine (3/3); thymic atrophy (3/3); petechial hemorrhages in proventriculus (1/3).
	Day 5 pi: severe conjunctivitis, severe depression: 3/5 were unable to stand; 1/5 had neurological signs and depression, 1/5 died overnight. All birds were euthanized *in extremis*.	
rZJ1-IL2	Day 1 pi: no signs	Day 2 pi: conjunctivitis (2/3); splenomegaly (3/3); hemorrhages in: cecal tonsils (2/3), bursa (1/3), and thymus (1/3).
	Day 2 pi: moderate conjunctivitis	Day 3 pi: conjunctivitis (3/3); mottled and enlarged spleen (3/3); diarrhea (1/3).
	Day 3 pi: moderate conjunctivitis and mild depression	Day 4 pi: severe bilateral conjunctivitis (3/3); enlarged and mottled spleen (3/3); necrosis in cecal tonsils (2/3); mild thymic atrophy (1/3).
	Day 4 pi: moderate to severe conjunctivitis, depression, and one animal (1/10) with neurological signs (unable to stand with head twitch)	Day 5 pi: conjunctivitis (2/3); bursal atrophy (3/3); thymic atrophy (1/3); enlarged and mottled spleen (1/3); mild hemorrhages in duodenal loop (1/3); cecal tonsils necrosis (1/3); severe dehydration (2/3).
	Day 5 pi: bilateral moderate to severe conjunctivitis, depression	
BHI	No lesions or clinical signs observed.	

Numbers indicate the fraction of birds presenting a certain clinical sign or lesion over the number of birds remaining in the cage (clinical signs), or the number of birds necropsied that day (lesions)

Clinical signs and mortality for each infection group are summarized in Table 1. In both groups, clinical signs consisted of various degrees of conjunctivitis and depression, which worsened until day 5 pi (end of the experiment). Similar to what was observed in the survival experiment, birds infected with rZJ1-IL2 displayed moderate to severe conjunctivitis earlier (day 2 pi) than those infected with rZJ1-GFP (day 3 pi). Birds infected with rZJ1-IL2 showed moderate depression at day 4 and 5 pi; however, no bird was terminally ill at the end of the experiment (day 5 pi). In rZJ1-GFP-infected birds, overall demise and severe depression became evident starting at day 4 pi and culminated at day 5 pi when one bird died spontaneously (1/5) and the rest (4/5) were euthanized *in extremis*.

Gross pathological findings for both experimental groups are presented in Table 1. Both rZJ1-IL2 and rZJ1-GFP caused similar lesions, which targeted mostly lymphoid organs and consisted of conjunctivitis, atrophy and necrosis of thymus, spleen, cecal tonsils and bursa of Fabricius (Table 1). Main differences among viruses consisted in the severity and extent of multifocal petechial intestinal hemorrhages at day 4 and 5 pi, which were severe and observed in six rZJ1-GFP-infected birds (Fig. 4, panel c), and mild and observed in only two rZJ1-IL2-infected birds (Fig. 4, panel d). Birds in the mock-infected group did not present any clinical signs or macroscopic lesions.

### Histopathology

**Fig. 5:**
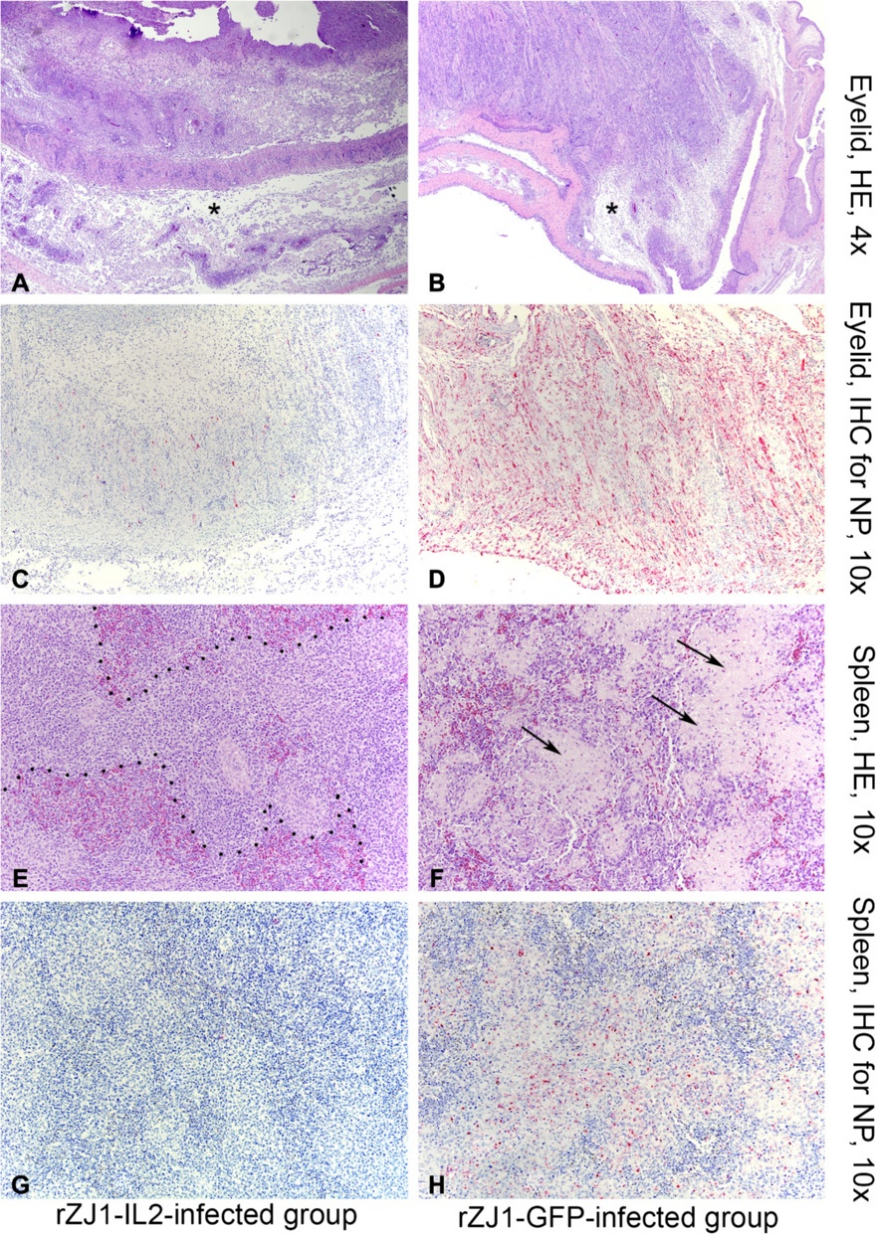
Photomicrographs illustrating hematoxylin and eosin staining (HE, first and third rows of panels) and immunohistochemistry (IHC, second and fourth rows of panels) on sections of eyelid (panels **a**-**d**) and spleen (**e**-**f**) at day 5 pi. Tissues were harvested from 4-week-old White Leghorn chickens infected with rZJ1-IL2 (first column of panels), and rZJ1-GFP (second column of panels). Alkaline phosphatase method and hematoxylin counterstain. In the eyelid, histopathological changes consist of severe edema (*asterisks*), which markedly expands the submucosa, accumulation of pleomorphic cellular infiltrate (macrophages, heterophils, lymphocytes), exudation of fibrin, and multifocal areas of coagulative necrosis. This severe conjunctivitis is similar in intensity in both rZJ1-Il2 (panel **a**) and rZJ1-GFP (panel **b**) groups. Presence of lesion in the eyelid is associated with positive immunohistochemical labeling, which is intense and diffuse in rZJ1-GFP-infected birds (panel **d**), while in rZJ1-IL2-infected birds is less intense and multifocal (panel **c**). In the spleen, rZJ1-IL2-infected birds display mild to moderate lymphoid depletion and accumulation of prominent macrophage in the ellipsoid areas, which appear confluent (*dashed lines*, panel **e**). These changes are associated with minimal NDV immunohistochemical labeling, which is not present at day 5 pi (panel **g**). Birds infected with rZJ1-GFP show severe lesions in the spleen, consisting of lymphoid depletion, prominent macrophages, exudation of fibrin and accumulation of necrotic debris (*arrows*, panel **f**). These lesions are associated with intense and diffuse immunohistochemical labeling for NDV (panel **h**)

Severity of histopathological lesions in selected organs is summarized in Table 2. Overall, lesions between the two groups had similar severity at day 2 and 3 pi, while significant differences became evident at day 4 and 5 pi, when lesions were markedly milder in rZJ1-IL2- compared to rZJ1-GFP-infected birds (Table 2). In both groups at days 2 and 3 pi, there was prominent conjunctivitis, with abundant infiltration of inflammatory cells and extensive edema that progressed at days 4 and 5 pi displaying fibrin exudation and necrosis (Fig. 5, panel a, b). At day 2 and 3 pi, in both rZJ1-IL2- and rZJ1-GFP-infected birds, the spleen showed prominent macrophages compared to mock-infected birds, resulting in overall increased size of the Schweigger-Seidel sheaths (splenic ellipsoids), which appeared almost confluent.

**Table 2:** Intensity of lesions (HE) and immunohistochemical staining (IHC) for NDV nucleoprotein in selected organs by days post infection (dpi). BHI-infected group did not present any lesions

		rZJ1-GFP			rZJ1-IL2		
Organs	DPI	3	4	5	3	4	5
Eyelid	HE	+++	+++	+	+++	+++	+++
	IHC	++	+++	++	++	++	+
Spleen	HE	++	++++	+++++	++	++	++
	IHC	++	+++	+	+	-	-
Thymus	HE	-	+++	+++	-	+	+++
	IHC	-	+++	++	+	+	-
Bursa of Fabricius	HE	+	+++	+++	+	+++	+++
	IHC	+	+++	++	-	+	-
Cecal Tonsils	HE	+	+++	+++	+	++	+
	IHC	+	+++	++	+	+	-
Lung	HE	-	++	++	-	-	-
	IHC	-	-	-	-	-	-
Heart	HE	-	+	-	-	-	-
	IHC	-	-	-	-	-	-
Brain	HE	-	+	+	-	+	+
	IHC	-	-	-	-	-	-

**Histological grading**

Eyelid: + presence of severe edema, which expands the submucosa; ++ multifocal inflammatory infiltrate; +++ coalescing / diffuse inflammatory infiltrate

Thymus: lymphoid depletion and apoptosis / necrosis of the thymic cortex, + < 20 %; ++ 26 to 50 %; +++ > 50 %

Cecal tonsil and Bursa: lymphoid depletion / necrosis, + < 20 %, ++ 26 to 50 %; +++ > 50 %

Spleen: prominent macrophages surrounding the penicillary arteries, +; confluent Schweigger-Seidel sheaths, ++; lymphoid depletion and apoptosis +++; diffuse necrosis and fibrin exudation ++++

Brain: vascular reactivity, +; perivascular mononuclear cuffing ++

**Immunohistochemical grading**

- = no IHC signal present

+ = rare cells in the section are positive on IHC

++ = positive cells seen, <50 % of all high power fields (HPF)

+++ = positive signal seen in > 50 % of HPF

At day 4 and 5 pi, rZJ1-IL2-infected birds displayed prominent and confluent Schweigger-Seidel (ellipsoids) sheaths, however no extensive lymphoid depletion or fibrin exudation was observed (Fig. 5, panel e). The cecal tonsils showed mild to moderate lymphoid depletion at day 4 and 5 pi. Bursa and thymus had more severe lesions, compared to the other lymphoid organs at day 5 pi, consisting of multifocal follicular lymphoid depletion in the bursa and severe diffuse lymphoid necrosis in the thymic cortex (prominent “starry sky” effect) associated with fibrin exudation (data not shown). By day 4 pi, rZJ1-GFP-infected birds presented very severe lesions in all the lymphoid organs, consisting of lymphoid depletion, necrosis, and multifocal fibrin deposition with greatest prominence in cecal tonsils and spleens. By day 5 pi, lesions consisting of lymphoid depletion, necrosis, and fibrin exudation also involved thymus and bursa and became progressively more severe in the spleen (Fig. 5, panel f).

In the brains of rZJ1-IL2-infected birds, at both day 4 (2/3 birds) and day 5 (2/3 birds) pi, there was accumulation of mononuclear cells within the perivascular spaces of scattered vessels (perivascular cuffing), mainly in periventricular regions and cerebellar peduncles. In the brain, at both day 4 and 5 pi, most birds inoculated with rZJ1-GFP (4/6 birds) did not show perivascular cuffing, but showed scattered glial nodules in the brainstem and multifocal vascular reactivity (plumped endothelial cells associated with few scattered mononuclear inflammatory cells within the vascular wall) in the periventricular areas.

### Immunohistochemistry for NDV nucleoprotein

To detect viral distribution in tissues, IHC for NDV nucleoprotein was performed on samples from rZJ1-IL2-, rZJ1-GFP-, and mock-infected birds. A summary of the distribution and intensity of immunohistochemical (IHC) staining for the NDV NP is presented in Table 2. Immunohistochemical labeling for the NDV NP was mainly confined to the lymphoid tissues. Considering all time points, birds inoculated with either virus had 5/5 positive lymphoid organs. However, IHC signal was markedly less intense and confined to few areas in the lymphoid organs of rZJ1-IL2- compared to rZJ1-GFP-infected birds, especially at day 4 and 5 pi. Immunolabelling for NDV NP was decreased in intensity in the eyelids of rZJ1-IL2- compared to rZJ1-GFP-infected birds, albeit lesions were comparable in severity (Fig. 5, panels c and d). Differences in immunolabelling were striking in the spleen, where minimal to no signal of NDV NP was observed in rZJ1-IL2-compared to rZJ1-GFP-infected birds (Fig. 5, panels g and h). Mock-infected birds did not present any immunoreactivity for NDV nucleoprotein.

### Co-expression of NDV NP and IL-2

**Fig. 6:**
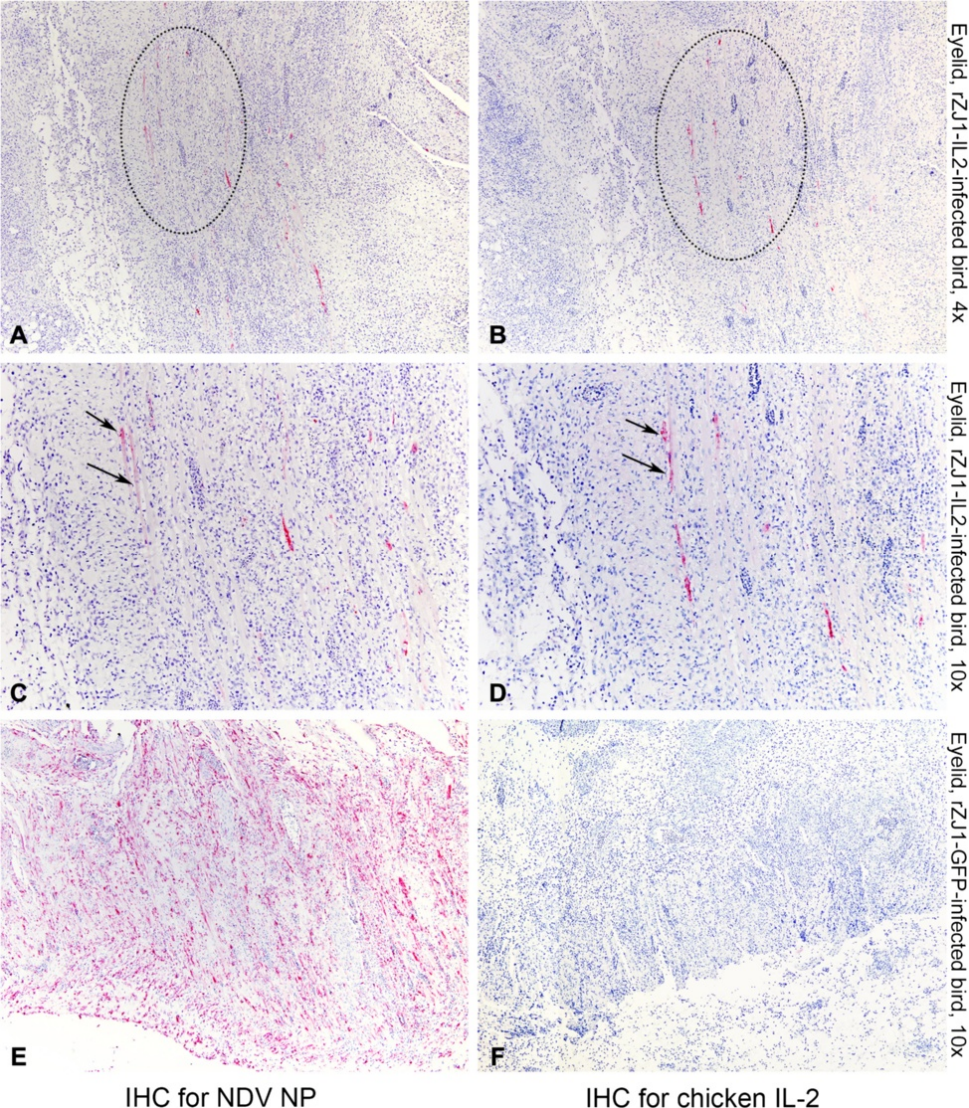
Photomicrographs illustrating immunohistochemistry (IHC) for NDV NP (first column of panels) and IL-2 (second column of panels) on sections of eyelids. Tissues were harvested from 4-week-old White Leghorn chickens infected with rZJ1-IL2 (**a**-**d**) and rZJ1-GFP (**e**, **f**) at day 5 pi. Alkaline phosphatase method and hematoxylin counterstain. At low magnification, in the eyelids of rZJ1-IL2-infected birds, the same areas that are immunolabeled for NDV NP are also positive for chicken IL-2 (*dotted circles*). At higher magnification, scattered cells in consecutive sections are immunolabeled for both NDV NP and IL-2 (*arrows*). No signal for IL-2 is observed in sections of eyelids from rZJ1-GFP-infected animals (**f**)

Sections of eyelid, which showed highest level of NDV replication by IHC, were chosen to assess IL-2 production by using IHC against chicken IL-2. Eyelids from rZJ1-IL2- and rZJ1-GFP-infected birds at day 4 and 5 pi were assayed for IHC for both NDV NP (Fig. 6, panels a, c, e) and chicken IL-2 (Fig. 6, panels b, d, f). Results showed that the all the eyelids of rZJ1-IL2-infected birds showed positive staining for IL-2 and NDV NP, while the eyelids of rZJ1-GFP-infected were negative for IL-2 and positive for NDV NP. In consecutive sections, NDV NP and IL-2 expression could be observed in the same areas, and occasionally in the same cells (in Fig. 6, compare panels a with b and c with d). These results show that IL-2 is expressed in the context of rZJ1-IL2 virus replication *in vivo*.

### Virus isolation and titration

**Fig. 7:**
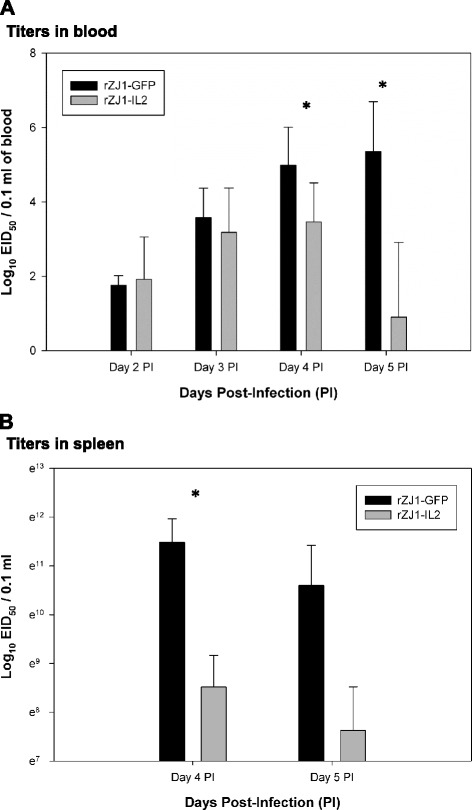
Titration of rZJ1-GFP and rZJ1-IL2 in whole blood (panel **a**) and spleens (panel **b**) of infected birds. *indicates significant difference between rZJ1-IL2- versus rZJ1-GFP-infected groups (*p* < 0.05). Bars represent standard deviation, *n* = 5

**Fig. 8:**
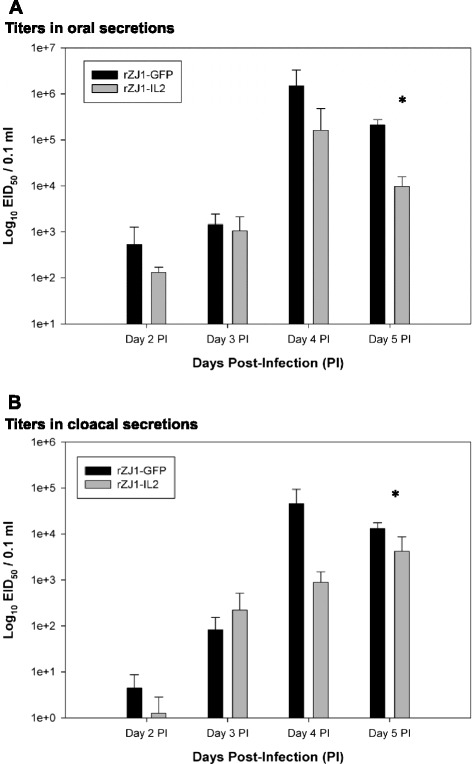
Titration of rZJ1-IL2 and rZJ1-GFP in oral (panel **a**) and cloacal (panel **b**) secretions of infected birds. *indicates significant difference between rZJ1-GFP- versus rZJ1-IL2-infected groups (*p* < 0.05). Bars represent standard deviation, *n* = 5

Systemic virus replication in infected birds was assessed by virus isolation and titration in whole blood (Fig. 7, panel a), spleens (Fig. 7, panel b), and in oral and cloacal secretions of rZJ1-IL2- and rZJ1-GFP-infected birds (Fig. 8). NDV was isolated via standard methods from tissues and swabs in embryonated chicken eggs, and titers were determined by limiting dilution and expressed as embryo infectious dose 50 % (EID_50_) per 0.1 ml [5]. Viremia from the rZJ1-IL2 group peaked at day 4 pi (mean, 10^3.46^ EID_50_ per 0.1 ml of blood) and drastically decreased at day 5 pi (mean, 10^0.9^ EID_50_ per 0.1 ml of blood) (Fig. 7, panel a). In rZJ1-GFP-infected birds, viremia progressed from day 2 to day 5 pi when it reached 10^5.35^ EID_50_ per 0.1 ml of blood (mean). Differences in virus titers were determined as statistically significant between rZJ1-IL2 and rZJ1-GFP groups at day 4 and 5 pi (*Mann–Whitney–Wilcoxon test*, *p* < 0.05).

Similarly, the amount of virus present in the spleen of infected birds at days 4 and 5 pi was lower in the rZJ1-IL2- than in rZJ1-GFP-infected birds. For rZJ1-IL2 and rZJ1-GFP groups respectively, at day 4 pi, titers in the spleens had an average of 10^3.7^ and 10^4.99^ EID_50_ per 0.1 ml of spleen homogenate, and at day 5 pi, titers were 10^3.31^ and 10^4.60^ (Fig. 6, panel b). Differences in virus titers were deemed statistically significant between rZJ1-IL2 and rZJ1-GFP groups at day 4 pi (*Mann–Whitney–Wilcoxon test*, *p* < 0.05).

Results of titration of oral and cloacal secretions showed that rZJ1-IL2 shed in lower quantities compared to rZJ1-GFP at all time points tested (Fig. 8, panels a and b). Shedding in oral secretions for both rZJ1-IL2 and rZJ1-GFP peaked at day 4 pi (mean, rZJ1-IL2: 10^5.21^, rZJ1-GFP: 10^6.18^ EID_50_ per 0.1 ml) and slightly decreased at day 5 pi (Fig. 8, panel a). In cloacal swabs, the highest amount of virus shed in the rZJ1-IL2 group was at day 5 pi (mean, 10^3.62^ EID_50_ / 0.1 ml), while in the rZJ1-GFP group, the highest titer was at day 4 pi (mean, 10^4.66^ EID_50_ / 0.1 ml) (Fig. 8, panel b). Differences in virus titers were statistically significant between rZJ1-IL2 and rZJ1-GFP groups at day 5 pi for both oral and cloacal swabs (*Mann–Whitney–Wilcoxon test, p* < 0.05).

## Discussion

In the present study, the effects of the chicken IL-2 gene expression during *in vivo* infection of SPF chickens by a virulent NDV were investigated. A virulent NDV virus expressing IL-2 was compared to an identical clone expressing the GFP gene. Since it has been shown that the insertion of additional nucleic acids in the genome may slightly affect the capacity of the virus to replicate [29], rZJ1-GFP was considered to be the most suitable control for rZJ1-IL2 in all animal experiments. This control has proven to be suitable for this experiment as no effect on viral replication was observed in highly controlled *in vitro* growth curves.

Animal experiments conducted in 4-week-old chickens demonstrated that expression of IL-2 by rZJ1-IL2 induced an attenuated phenotype compared to rZJ1-GFP, resulting in decreased severity of hemorrhagic lesions in the intestine, and decreased severity of lymphoid depletion and necrosis in multiple lymphoid organs. Thus, as it would be expected by an effect produced on the host by a gene expressed during viral replication, these differences were more pronounced in tissues that are normally targeted by velogenic NDV [6], i.e., the lymphoid organs, which also have a higher numbers of possible effector cells that can be stimulated by IL-2.

The observed differences in pathology were accompanied with marked differences in the magnitude of tissue viral load (titers) between rZJ1-IL2- and rZJ1-GFP-infected birds. As shown by IHC, intensity of immunolabelling paralleled the severity and distribution of lesions, being less intense and less distributed in the organs of rZJ1-IL2- compared to rZJ1-GFP-infected birds.

The observed decreased viral load in different tissues suggests that IL-2 may have a general antiviral effect against NDV; however, the nature and extent of these effects await further investigation. The pleiotropic effects of IL-2 in mammals have been extensively reviewed [31], however, data in avian species are limited. In mammals, IL-2-dependent activation of effector cells has been proven important to control infection caused by viruses, such as HSV-1 [15, 16], cytomegalovirus [17], and lymphocytic choriomeningitis virus (in the persistent infection phase) [32]. Similar to what is described in the present study, there are several reports in the literature documenting attenuation of recombinant viruses expressing IL-2 in mice. For instance, a recombinant strain of HSV-1 expressing IL-2 was markedly attenuated in mice compared to wild-type parental strains, most likely due to activation of CD8+ and CD4+ effector T lymphocytes [15]; when expressed by a recombinant vaccinia virus, IL-2 was able to dramatically reduce morbidity and mortality in nude mice through upregulation and enhanced expression of IFN-γ by NK cells [18, 19]; a recombinant hRSV strain expressing IL-2 was moderately attenuated in respiratory tract of infected mice and induced upregulation of IFN-γ in lung mononuclear cells [20].

Results of the present study showed that IL-2 attenuates the severity of lesions and virus distribution when expressed simultaneously with replication of a virulent NDV strain, suggesting a possible indirect antiviral effect of IL-2 against NDV. Similarly to the studies reported above, an antiviral effect of IL-2 against NDV could have been the direct consequence of the activation of effector cells, such as NK cells, or indirect production of more potent antiviral cytokines, such as IFN-γ. It is possible that a more pronounced effect of IL-2 gene may not have been detected in our study because of the highly virulent characteristics of rZJ1 strain, which usually induces a disease course of only of a few days (usually up to 5 [6]). This timeframe may have been too short to yield a polarized Th1 response or to activate cytotoxic T cells (all functions of IL-2 [33]).

It is possible that the antiviral mechanisms observed in mammals could also develop in NDV-infected chickens, since in this species IL-2 has similar roles as in mammals. In fact, chicken IL-2 is produced by stimulated splenic lymphocytes, induces proliferation of lymphocytes [22, 24], contributes to differentiation/activation of chicken regulatory T cells (T-regs) and NK cells [33, 34], and is implicated in the development of cell-mediated immunity [35, 36]. Cell-mediated immunity and NK cell activation, in turn, could avail NDV clearance [37]. It is possible that expression of IL-2 could have determined upregulation of other cytokines with more prominent antiviral activity, such as IFN-γ. For instance, in mice, IL-2 is able to induce IFN-γ production in peritoneal macrophages [38]. In a previous study, we have shown that expression of IFN-γ by the same NDV strain used in the present work (rZJ1-IFNγ), induced a dramatic decrease in morbidity and mortality of infected birds [26], suggesting that IFN-γ has a more potent effect in controlling NDV infection than IL-2. Further studies are necessary to elucidate the transcriptional activity of chicken tissues infected with rZJ1-IL2.

## Conclusion

In summary, our results show that production of IL-2 simultaneously with replication of a virulent NDV strain was sufficient to reduce systemic (blood) and localized (tissues) viral loads, however it was not sufficient to decrease mortality of infected chickens. These data are in agreement with observations made with other viral pathogens and suggest that IL-2 may work with other cytokines to control NDV infection *in vivo*. As it would be expected, the results suggest that expression of a single gene with antiviral properties may not be sufficient to substantially alter the outcome of infection caused by a highly virulent virus with a rapid disease course. Nonetheless, IL-2 partially decreased the pathogenicity of a velogenic NDV strain by lessening tissue damage and systemic viral load, suggesting that IL-2 in conjunction with other genes may have a greater attenuating effect with less-virulent NDV pathotypes (i.e., mesogenic or lentogenic strains), and therefore warranting further tests with these strains.

## Materials and methods

### Cells and viruses

DF-1 cells (Chicken embryo fibroblast cell line; ATCC CRL 12203) and HEp-2 cells (American Type Culture Collection, ATCC, Manassas, VA, CCL-23) were cultured in Dulbecco’s modified Eagle’s medium (DMEM) (with 5 % fetal bovine serum (FBS) and 1 % streptomycin / penicillin) at 37 °C with 5 % CO_2_. HD-11 cells (chicken bone marrow macrophages cell line [39]) were cultured in Roswell Park Memorial Institute (RPMI) media (with 10 % FBS with 1 % streptomycin/penicillin at 37 °C with 5 % CO_2_). The recombinant modified *Vaccinia* virus Ankara expressing the T7 RNA polymerase (a generous gift of Bernard Moss, National Institutes of Health) was grown in primary chicken embryo fibroblast cells. The recombinant NDV strains, rZJ1-GFP and rZJ1 were rescued from full-length clone plasmids as previously described [26, 29].

### IL-2 cloning

The open reading frame (ORF) of chicken IL-2 cloned into pCR2.1 was a generous gift of Dr. Sundick (Wayne State University, MI). The IL-2 ORF was modified as follows: a Kozak sequence (GCCGCCACC) was added before the start codon (ATG); the nucleotide immediately after the first ATG codon was mutated from an adenosine to a guanidine residue (A → G), resulting in a substitution of a methionine to a valine in the amino acid sequence; a stretch of 6 histidine amino acids was added at the carboxy-terminal of the protein. This modified IL-2 ORF was previously shown to be expressed in eukaryotic systems and to be biologically active in chickens [22, 24]. For insertion within the rZJ1 genome, the “gene start” (GS), “gene end” (GE), and ApaI restriction site sequences were added to the IL-2 ORF by PCR amplification (High Fidelity PCR kit, Promega, Madison, WI) using primers IL2-F (5′-ctgggccctcttagaaaaaatacgggtagaagtaccggatccgccgccaccatgg-3′) and IL2-R (5′-ggccggttgggccctctcctcaatgatgatgatgatgatgtttttgcagatatctca-3′), as previously described [26, 29]. Amplicons were cloned into a TOPO vector and the resulting plasmid was named pCRIL2. The number of nucleotides between the two ApaI sites flanking the whole insert (IL-2 ORF, GS, GE) is an exact multiple of six (504 bases).

### Construction of recombinant cDNA clones of rZJ1-IL2 and rZJ1-GFP

The strategy for subcloning the modified IL-2 ORF from the pCRIL2 plasmid into the full-length clone of NDV strain ZJ1 (TVT7 expression vector) was previously described and was adopted in this study [26, 29]. The resulting full-length clone plasmid was designated pNDV/ZJ1-IL2.

### Rescue of the viruses

Recombinant virus rZJ1-IL2 was rescued from pNDV/ZJ1-IL2 plasmid in Hep-2 cells, as previously described [26, 40]. From the allanotic fluid of infected eggs, RNA was extracted and the region corresponding to positions 2857–3676 of the NDV ZJ1 genome was amplified by RT-PCR and sequenced to confirm presence of the IL-2 insert. The other two viruses used in this study, rZJ1-GFP and rZJ1 were previously plasmid-rescued and characterized [26]. The NDV strain rZJ1 is the parental strains, which was used to produce a full-length clone plasmid and to insert the green fluorescent protein (GFP) gene into the genome (rZJ1-GFP) [29]. The GFP gene is inserted in the same location of the IL-2 insert in rZJ1-IL2.

### Growth curves

The replication kinetics of rZJ1-IL2, rZJ1-GFP, and rZJ1 were compared by multi-step growth curves in DF-1 and HD-11 cells. Cells were plated at 1×10^6^ (DF-1) or 7×10^5^ (HD-11) cells / well in 6 well plates and were infected with each NDV virus at a MOI = 0.01 the following day. Briefly, maintenance media was discarded and cells were incubated with virus inoculum diluted in reduced-serum media (1 % serum, 1 ml / well) for 1 h at 37 °C with 5 % CO_2_. After adsorption, inoculum was removed, cells washed three times with PBS, and complete growth media (supplemented with 10 % serum) was added. At 0, 6, 12, 24, 36, 48, and 72 h post infection (hpi), 200 μL of supernatant were harvested for virus titration and replaced with 200 μL of fresh media. Virus titers in the supernatant were determined by limiting dilution in DF-1 cells in 96-well plates and expressed as tissue culture infectious dose 50 % (TCID_50_) per ml using the Spearman-Karber method. Each growth curve was conducted in triplicate.

### Eggs and chickens

The Southeast Poultry Research Laboratory (SEPRL) specific pathogen free (SPF) White Leghorn flock was the source for all embryonated chicken eggs and chickens. All birds were housed in negative pressure isolators under biosafety level (BSL)-3 enhanced (E) conditions at SEPRL and provided food and water *ad libitum*. Virus propagation, isolation, and titration were conducted in nine to ten-day-old SPF embryonated eggs, by inoculation of infectious material in the chorioallantoic cavity [5]. Titration was carried out by limiting dilution in eggs, positive eggs were assessed by hemagglutination assay and results were expressed as embryo infectious dose 50 % (EID_50_) [5].

### Expression of IL-2 *in vivo* and *in vitro*

The ability of rZJ1-IL2 to express chicken IL-2 was assessed by western blot (WB) in the supernatant and lysate of DF-1 cells, as well as in the allantoic fluid of embryonated eggs. DF-1 cells were plated at 5x10^5^ cells/well and infected the following day with rZJ1-GFP or rZJ1-IL2 at a MOI = 1. After 48 h, cell supernatant was collected and cells were harvested by adding Laemli buffer supplemented with 10 % β-Mercaptoethanol (lysis buffer) directly onto the cells. Allantoic fluid was extracted at 48 hpi from eggs inoculated with 10^3^ EID_50_ of rZJ1-IL2 or rZJ1-GFP in BHI media. Lysis buffer was mixed 1:1 with allantoic fluid or cell supernatant. All cell lysates were then incubated for 5 min at 100 °C and sonicated for 10 s at 70 % amplitude with a cup sonicator (Branson, Dandury, CT). After separation in 12 % SDS-PAGE (pre-cast gels [Biorad, Hercules, CA]) at 150 V, proteins were transferred by electrophoresis to a nitrocellulose membrane (semi-dry transfer, 30 min at 20 V). After transfer, membranes were blocked for 1 h at room temperature (RT) with PBST (Phosphate-buffered saline, supplemented with 0.1 % Tween) containing 5 % bovine serum albumin (BSA). After blocking, primary antibodies for IL-2 (Polyclonal rabbit, Anti-chicken IL-2 [King Fisher, Carlsbad, CA]) and NDV nucleoprotein (in-house produced, hybridoma mouse monoclonal, clone 96–11) were diluted 1:3000 and 1:1000 respectively in blocking buffer and applied for 1 h at RT with constant shaking. Blots were then washed with PBST and incubated for 1 h at RT with secondary antibodies applied at 1:5000 dilutions (alkaline phosphatase-conjugated anti-rabbit or anti mouse [Santa Cruz, Dallas, TX]). Blots were then washed three times with PBST and incubated with chromogenic substrate (NBT/BCIP, Roche) until signal developed.

### Intracerebral Pathogenicity index test (ICPI)

ICPI test was performed according to standard protocols [3, 4]. Chickens were inoculated intracerebrally at one-day of age with 0.05 ml of a 1:10 dilution of filtered infective allantoic fluid from rZJ1-IL2-infected eggs. Chickens were monitored daily and scored as normal, sick or paralyzed, and dead to compile a score over an 8-day observation period. The final scores for each virus were tallied on a scale from 0 to 2.

### Survival curves and clinical signs

To assess the ability of rZJ1-IL2 and rZJ1-GFP to elicit clinical signs and cause death of infected birds, 4-week-old White Leghorn chickens were randomly assigned to seven experimental groups of 8–10 birds (rZJ1-IL2 groups at medium and high doses had 10 birds, the other groups had 8 birds), with each group allocated in a single isolator. Birds from each group were infected with a suspension of BHI containing either rZJ1-IL2 or rZJ1-GFP at three different target doses: 10^4^ (low), 10^5.5^ (medium), and 10^6.5^ (high) EID_50_ / bird. Half of the inoculum (0.05 ml) was delivered in the left conjunctival sac and the other half was delivered in the choanal slit. A BHI-inoculated group served as the negative control. Birds were observed for clinical signs every day and daily deaths for each group were recorded to tally a final survival curve for each virus at the given concentration. Birds that were *in extremis* at the time of observation were humanely euthanized and counted as dead for the next day.

### Pathogenesis assessment in chickens

Sixty, 4-week-old SPF White Leghorn chickens were randomly allocated in three experimental groups of 20 birds each and inoculated with a BHI suspension of rZJ1-IL2, rZJ1-GFP, or BHI alone (mock-infected). The target dose of the inoculum was 10^5.5^ TCID_50_ and it was applied with the same methods used for the survival curves. Birds were monitored daily for 5 days, and starting from day 1 post-infection (pi), 5 birds from each group were euthanized and spleens, blood, oropharyngeal and cloacal swabs were collected for virus isolation and titration. Additionally, the following tissues were harvested for histopathology from three out the five euthanized birds: eyelids, spleen, thymus, bursa of Fabricius, cecal tonsils, heart, brain, and liver. At each day, birds *in extremis* or presenting more pronounced clinical signs were sampled first for human reasons. Tissues were fixed in 10 % neutral buffered formalin for 52 h. All sampled tissues were routinely embedded in paraffin and 3 μm sections were cut for hematoxylin and eosin staining (HE) and immunohistochemistry (IHC). The scoring system for assessment of microscopic lesions was previously published [41], and is reported in the caption of Table 2.

### Immunohistochemistry

To detect viral distribution in tissues, IHC was performed on samples from rZJ1-IL2-, rZJ1-GFP, and mock-infected birds, as previously described [41]. Briefly, slides were deparaffinized, rehydrated, microwaved for 20 min at minimum power in Vector antigen unmasking solution (Vector Laboratories, Burlingame, CA) and blocked with an universal blocking reagent (Biogenex, San Ramon, CA). The primary antibody (polyclonal) was raised in rabbit against a synthetic peptide (TAYETADESETRRIC), which is highly conserved in NDV nucleoprotein, and used at 1:8000 dilution [42]. The detection system was an avidin–biotin–alkaline phosphatase system (Vector Laboratories, Burlingame, CA) coupled with a naphthol-based chromogen (Fast Red, Dako, Carpinteria, CA). Sections were counterstained lightly with hematoxylin and coverslipped with Permount for a permanent record. The scoring system for assessment of immunolabeling for NDV NP was previously published [41], and is reported in the caption of Table 2. For IL-2, the same procedure was deployed; the chicken IL-2 antibody was a rabbit polyclonal (Kingfisher, Carlsbad, CA) used at 1:1000 dilution.

### Virus isolation and titration

Oral and cloacal swabs obtained from each bird were placed in separate tubes containing 1.5 ml of brain-heart infusion broth (BHI) with antibiotics (2000 U/ml penicillin G, 200 μg/ml gentamicin sulfate, and 4 μg/ml amphotericin B; Sigma Chemical Co., St. Louis, MO). Swab sample tubes were centrifuged at 1000 × g for 20 min and the supernatant removed for virus isolation and titration in eggs, according to standard procedures [5, 43].

Harvested spleens were homogenized with a Stomacher in a 10 % w/v solution with PBS supplemented with antibiotics (2000 U/ml penicillin G, 200 μg/ml gentamicin sulfate, and 4 μg/ml amphotericin B [Sigma Chemical Co., St. Louis, MO]). For each sample, virus isolation was performed by inoculating undiluted homogenized samples into three embryonated eggs (200 μL per egg). Virus titer in positive eggs was assessed in embryonated eggs, as per standard procedures [5, 43].

Whole blood from chickens was harvested into EDTA-containing tubes and 200 μL were inoculated into three SPF embryonated eggs per sample for virus isolation (VI). Virus titers from VI-positive samples were subsequently assayed in embryonated eggs [5, 43].

### Data analysis

Statistical analysis for growth curves of rZJ1-IL2 and rZJ1-GFP was carried out using a Two-Way ANOVA, followed by a Tukey test for multiple comparisons. A Log-Rank test with multiple comparison between groups was used for the survival curves. A non-parametric *t-test* (Mann–Whitney–Wilcoxon test) between rZJ1-IL2 and rZJ1-GFP groups was performed to assess the statistical differences in virus titers in spleen, blood, and swabs at the same time point. For each test, significance was considered with *p* < 0.05. Statistical software was JMP version 8.0 (SAS Institute, Raleigh, NC). Survival curves were analyzed with GraphPad Prism (GraphPad Software, San Diego California USA).

## Additional file

Additional file 1: Table S1. Summary of clinical signs and mortality in 4-week-old chickens infected with rZJ1-GFP, rZJ1-IL2, and BHI. Numbers in the cells represent the percentages of birds presenting the clinical sign over the total birds present in the group at the time. Highlighted cells represent groups where all the animals are dead. (XLSX 13 kb)

## Abbreviations

ND: Newcastle disease
NDV: Newcastle disease virus
IL-2: Interleukin-2
ICPI: Intracerebral pathogenicity index
WB: Western blot
IHC: Immunohisotchemistry
HE: Hematoxylin and eosin
TCID50: Tissue culture infectious dose 50 %
EID50: Embryo infectious dose 50 %
SPF: Specific pathogen free
BHI broth: Brain-heart infusion broth.
